# Hypertriglyceridemia-Induced Acute Pancreatitis, Euglycemic Diabetic Ketoacidosis and COVID-19 Infection in a Patient With Type 2 Diabetes Taking a Sodium-Glucose Cotransporter 2 Inhibitor

**DOI:** 10.7759/cureus.19828

**Published:** 2021-11-23

**Authors:** Bernardo A Acevedo-Mendez, Yuting Ye, Negin Hajizadeh, Alyson Myers

**Affiliations:** 1 Department of Medicine, Northwell Health, Manhasset, USA; 2 Department of Medicine, North Shore University Hospital, Manhasset, USA

**Keywords:** covid-19, sodium-glucose cotransporter 2 inhibitors, euglycemic diabetic ketoacidosis, acute pancreatitis, hypertriglyceridemia

## Abstract

Recent landmark trials have increased the use of sodium-glucose cotransporter 2 inhibitors (SGLT-2i) in patients with type 2 diabetes (T2D). A rare but serious side effect of SGLT-2i is euglycemic diabetic ketoacidosis (euDKA), which usually occurs in the setting of acute illness such as the coronavirus disease 2019 (COVID-19).

We report a distinctive case of a patient with hyperlipidemia and T2D on SGLT-2i therapy who presented with hypertriglyceridemia-induced pancreatitis (HTGP) concurrently with euDKA and COVID-19. The patient’s initial labs included venous blood gas pH of 7.27, a blood glucose level of 146 mg/dL, serum triglyceride (TG) greater than 8,300 mg/dL and lipase of 527 U/L. Viral polymerase chain reaction (PCR) result for severe acute respiratory syndrome coronavirus 2 (SARS-CoV-2) was also positive. We suspect this patient has a primary disorder of lipoprotein metabolism which was exacerbated by stress from euDKA and COVID-19 infection. The patient was treated with intravenous fluids, fasting and intravenous insulin infusion. Resolution of euDKA and improvement of hypertriglyceridemia to less than 1,000 mg/dL occurred by day 6 and the patient was transitioned to subcutaneous basal-bolus insulin. On discharge, the SGLT-2i was discontinued and the patient was discharged on insulin, metformin, omega-3 fatty acids, and fenofibrate.

## Introduction

The use of sodium-glucose cotransporter 2 inhibitors (SGLT-2i) in patients with type 2 diabetes (T2D) has become more prevalent as benefits of cardiovascular and kidney outcomes were demonstrated in recent trials [1,2]. One rare but serious adverse effect of SGLT-2i is euglycemic diabetic ketoacidosis (euDKA), which is usually precipitated by acute illness, reduced food and fluid intake, reduced insulin doses, or use of alcohol [3]. A recent case series of five patients highlights that infection with severe acute respiratory syndrome coronavirus 2 (SARS-CoV-2) can precipitate EuDKA in patients taking SGLT-2i [4].

Here, we report a distinctive case of a patient with T2D and hyperlipidemia on SGLT-2i who presented with hypertriglyceridemia-induced pancreatitis (HTGP) concurrently with euDKA and coronavirus disease 2019 (COVID-19).

## Case presentation

A 43-year-old patient with a past medical history of gestational diabetes with progression to T2D without complications, hyperlipidemia, and body mass index (BMI) of 29.6 presented to our emergency department (ED) with right upper quadrant abdominal pain for two days, accompanied with nausea and vomiting. The patient denied a history of alcohol use. Home medications included metformin, empagliflozin, sitagliptin, and fenofibrate which were not taken consistently for the past 2-3 days due to nausea and vomiting. Of note, the patient was diagnosed with COVID-19 infection 12 days prior and received monoclonal antibody infusion 11 days prior to presentation. On presentation to ED viral polymerase chain reaction (PCR) result for SARS-CoV-2 was still positive. Vital signs on presentation were temperature 37 degrees Celsius, heart rate 119 beats per minute, blood pressure 139/94 mmHg, respiratory rate 20 breaths per minute and oxygen saturation 95% on room air.

**Figure 1:**
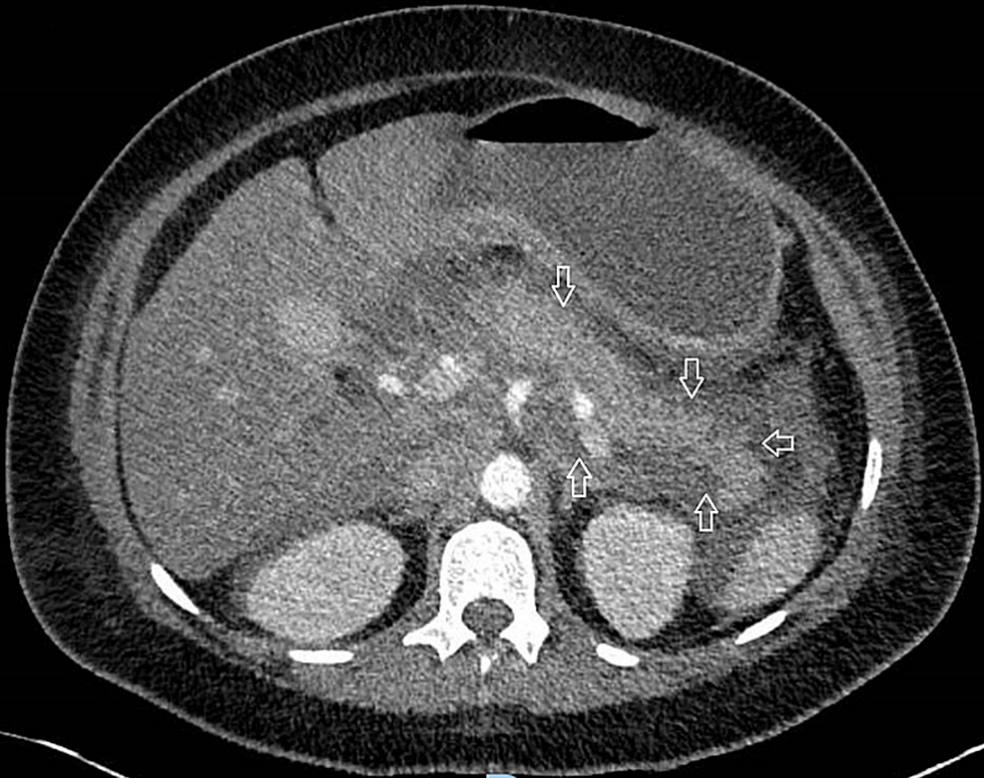
CT scan of the abdomen and pelvis Peripancreatic fat is infiltrated (arrows) and there is a fluid surrounding the pancreas and along with the lesser sac consistent with acute pancreatitis.

Initial labs were consistent with euDKA and very severe hypertriglyceridemia as shown in Table 1. Pregnancy test was negative and hemoglobin A1c (HbA1c) was 7.7% (61 mmol/mol). Based on symptoms and laboratory results HTGP was diagnosed. This was confirmed by imaging as CT abdomen and pelvis with IV contrast showed: “peripancreatic fat is infiltrated and there is fluid surrounding the pancreas and along the lesser sac,” as seen in Figure 1. In the ED, the patient was made nil per os (NPO) and home oral antihyperglycemic medications were discontinued. Fluid resuscitation with lactated ringer and intravenous insulin infusion were initiated and continued upon admission to the medical ICU. She was started on prophylactic enoxaparin in the setting of COVID-19 infection in a hospitalized patient as per our institution's protocols. The anion gap closed, and the acidosis resolved after two days. Serum triglycerides decreased to less than 1,000 mg/dL on day 6 of hospitalization, allowing for transition to subcutaneous basal-bolus insulin. The hospital course was complicated by volume overload requiring diuretics. The patient left against medical advice after eight days of hospitalization. Sitagliptin and empagliflozin were both discontinued at discharge. The discharge medication regimen included subcutaneous basal-bolus insulin, metformin, omega-3 fatty acids and fenofibrate.

**Table 1:** Initial labs of the patient

Test	Result
Plasma glucose level (mg/dL)	146
Venous blood gas pH	7.27
Venous blood gas pCO2 (mmHg)	42
Bicarbonate (mmol/L)	15
BUN (mg/dL)	8
Anion gap (mmol/L)	17
Beta-hydroxybutyrate (mmol/L)	4.1
Hgb A1c (%, mmol/mol)	7.7, 60.7
Triglycerides (mg/dL)	>8300
Lipase (U/L)	527
Calcium (mg/dL)	7.9
Albumin (g/dL)	3.2
AST (U/L)	19
ALT (U/L)	<5
Total bilirubin (mg/dL)	0.3
Urine pregnancy test	Negative

 

## Discussion

We describe a distinctive case of a patient with T2D on SGLT-2i who developed euDKA and HTGP shortly after acute COVID-19 infection. Hypertriglyceridemia is defined as fasting serum triglyceride levels above 150 mg/dL and is divided into four categories based on serum triglyceride levels: mild, 150-199 mg/dL; moderate, 200-999 mg/dL; severe, 1,000-1,999 mg/dL; and very severe, >2,000 mg/dL [5]. When serum triglyceride levels are > 1,000 mg/dL, the risk of developing acute pancreatitis is increased significantly [5]. Causes of hypertriglyceridemia include primary (genetic) and secondary disorders of lipoprotein metabolism, and a combination of both.

Considering the very severe hypertriglyceridemia (initial serum triglyceride > 8,300 mg/dL), our patient likely has an undiagnosed primary (genetic) disorder of lipoprotein metabolism. Three types of primary hyperlipoproteinemia (Types I, IV and V) are associated with significant hypertriglyceridemia [6]. Type I dyslipidemia is associated with elevated levels of chylomicrons, type IV dyslipidemia with elevated levels of very-low-density lipoprotein (VLDL), and type V dyslipidemia with elevated levels of VLDL and chylomicrons [6]. Type I dyslipidemia, also known as familial chylomicronemia, is caused by an autosomal recessive trait of lipoprotein lipase deficiency. Type I dyslipidemia is typically appreciated early in infancy and causes pancreatitis without exacerbating environmental factors [7,8]. It is unlikely that our patient has type I dyslipidemia. On the other hand, Type IV, also known as familial combined hyperlipidemia, and type V, also known as primary mixed hypertriglyceridemia, are both complex genetic disorders that usually present in adulthood and only cause pancreatitis with exacerbating environmental factors [7,8]. Our patient likely has type IV or V dyslipidemia and with exacerbating stress of euDKA and COVID-19 infection, subsequently developed HTGP.

 EuDKA was first described in 1973 in a case series of 37 young patients with type 1 diabetes (T1D) who presented with severe ketoacidosis with plasma bicarbonate of 10 mEq/L or less and blood glucose level of less than 300 mg/dL [9]. EuDKA is commonly seen in patients with T2D taking SGLT-2i, and serum glucose is typically less than 250 mg/dL [3]. The mechanism of SGLT-2i induced euDKA is as follows. SGLT-2i causes profound glucosuria which decreases plasma glucose levels and leads to increased glucagon secretion [10]. As glucose is the chief stimulus for insulin release, plasma insulin level decreases significantly [10]. On the other hand, plasma glucagon level increases because of decreased insulin secretion, and possibly also because of decreased SGLT-2 mediated glucose transport into α-cells [11]. Low insulin to glucagon ratio stimulates lipolysis and enhances lipid oxidation causing ketoacidosis [10]. Insulin plays a central role in the regulation of lipid metabolism. Diabetic ketoacidosis (DKA) is known to be a cause of hypertriglyceridemia [12]. Insulin deficiency causes an increase in lipolysis in adipose tissue with increased release of fatty acids. Increased delivery of free fatty acids to the liver leads to increased output of VLDL. Insulin deficiency also causes decreased activity of lipoprotein lipase which hydrolyzes triglycerides in lipoproteins [12]. The combination of increased serum VLDL and decreased activity of lipoprotein lipase result in hypertriglyceridemia, and when combined with hereditary disorders of lipoprotein metabolism can cause severe hypertriglyceridemia (serum triglyceride level > 1,000 mg/dL), causing acute pancreatitis [13]. The relationship between EuDKA and HTGP is not well studied; though considering the similar pathway of EuDKA and DKA in generating ketoacidosis, we suspect there is a connection between EuDKA and HTGP.

The breakdown of triglycerides into toxic free fatty acids by pancreatic lipases leads to inflammatory reactions resulting in pancreatic auto-digestion [14]. Research has shown that the level of serum triglyceride is positively correlated with the severity of acute pancreatitis [15]. Acute management of HTGP involves the reduction of triglycerides and treating pancreatitis. This is usually done with fasting status, intravenous fluids, continuous intravenous insulin infusion, fibrates, and pain control. Plasmapheresis can be considered in cases of multi-organ failure, worsening systemic inflammation, or lactic acidosis, though clinical benefits are not clearly established [13]. Long-term management focuses on preventing recurrence of HTGP by maintaining serum triglyceride levels less than 500 mg/dL, and according to newer studies, less than 200 mg/dL [16,17]. Long-term therapies include weight loss, limiting concentrated sugars and dietary fat, optimal control of diabetes, and a combination of lipid-lowering medications including fibrates, statins, niacin, and omega-3 fatty acids.

The limitations of this case were: the lack of genetic testing for disorders of lipid metabolism and type 1 diabetes (T1D) workup. The work-up for T1D was deferred as the patient was fairly well controlled on non-insulin agents.

## Conclusions

This case highlights the risks of SGLT-2i use in patients with T2D who acquire COVID-19 infection. SGLT-2i-associated euDKA can contribute to hypertriglyceridemia and severe hypertriglyceridemia can induce acute pancreatitis. Diagnosis of EuDKA is frequently missed or delayed due to the serum glucose being less than 250 mg/dL. It is important for physicians to have a high clinical suspicion of EuDKA in patients who are on SGLT-2i in the setting of acute COVID-19 illness. In addition, to consider that EuDKA can precipitate HTGP. In both conditions, the early initiation of continuous intravenous insulin infusion can improve outcomes.
